# Cloning and functional analysis of the molting gene *CYP302A1* of *Daphnia sinensis*

**DOI:** 10.1186/s12983-023-00483-2

**Published:** 2023-01-12

**Authors:** Huiying Qi, Huijuan Cao, Yajie Zhao, Yaqin Cao, Qide Jin, Yeping Wang, Kun Zhang, Daogui Deng

**Affiliations:** School of Life Science, Huaibei Normal University, Huaibei, 235000 People’s Republic of China

**Keywords:** *D. sinensis*, Ecdysone, *CYP302A1* gene, Cloning, Functional analysis

## Abstract

**Background:**

Molting is an important physiological process in the growth and development of arthropoda, which is mainly regulated by juvenile hormone and ecdysone. CYP302A1 is a key enzyme which plays a critical role in the synthesis of ecdysone in insects, but it has not been identified in cladocera.

**Results:**

The *CYP302Al* gene of *Daphnia sinensis* was cloned and its function was analyzed in this paper. The *CYP302Al* gene of *D. sinensis* was 5926 bp in full-length, with an open reading frame (ORF) of 1596 bp that encoded 531 amino acids (aa), a molecular weight of 60.82 kDa and an isoelectric point of 9.29. The amino acid sequence analysis revealed that there were five characteristic conserved regions of cytochrome P450 family (namely helix-C, helix-K, helix-I, PERF and heme-binding). In dsRNA mediated experiment, the expression level of *CYP302A1* gene decreased significantly (knock-down of 56.22%) in the 5% *Escherichia coli* concentration treatment. In addition, the expression levels of *EcR* and *USP* and *HR3* genes in the downstream were also significantly decreased, whereas that of *FTZ*-f1 gene increased significantly. In the 5% *E. coli* treatment, the molting time at maturity of *D. sinensis* prolonged, and the development of embryos in the incubation capsule appeared abnormal or disintegrated. The whole-mount in situ hybridization showed that the *CYP302A1* gene of *D. sinensis* had six expression sites before RNA interference (RNAi), which located in the first antennal ganglion, ovary, cecae, olfactory hair, thoracic limb and tail spine. However, the expression signal of the *CYP302A1* gene of *D. sinensis* disappeared in the first antennal ganglion and obviously attenuated in the ovary after RNAi.

**Conclusion:**

The *CYP302A1* gene played an important role in the ecdysone synthesis pathway of *D. sinensis*, and the knock-down of the gene affected the molting and reproduction of *D. sinensis*.

## Background

During the life history of cladocera (e.g. *Daphnia*), their growth and molting are alternately [1]. The molting action runs through their whole life cycle, and it is a necessary step before they grow and reproduce [2]. Molting is a result of long-term evolution in arthropod, which are regulated by many factors [3]. In crustacean ecdysis, ecdysteroid is the most important regulatory factor, which the expression levels vary among species [4]. Among ecdysones, 20-hydroxyecdysone (20E) is one of the more active hormones in insects [5]. Moreover, ecdysone can not only regulate the molting physiology in arthropods, but also plays important roles in their growth, reproduction and phenotypic plasticity [6–10].

The synthetic pathway of ecdysone has been extensively studied in insects [11, 12]. Usually, the synthesis of insect ecdysone is divided into two stages. Firstly, the cholesterol in food was digested and absorbed through the intestine, and then transported to the prothymus (PG) by hemolymph. The cholesterol was transformed to 5β-diketol (3D2, 22, 25dE) under the catalysis of both *Neverland* and *CYP307A1* gene [13–15]. Secondly, the 5β-diketol was converted to inactive ecdysterone catalyzed by various cytochrome P450s (*CYP306Al*, *CYP302Al*, and *CYP315Al*) [16–19]. The inactive ecdysone could be also converted to 20 E under the catalysis of the *CYP314A1* gene [20]. Among them, the *CYP307A1* (*Spook*, *Spo*), *CYP306A1* (*Phantom*, *Phm*), *CYP302A1* (*Disembodied*, *DIB*), *CYP315A1* (*Shadow*, *Sad*) and *CYP314A1* (*Shade*, *Shd*) genes are referred to as the Halloween genes. The 20E mediates its biological activities through the ecdysone receptor (EcR) complex, a heterodimer consisting of two nuclear hormone receptors, EcR and the retinoid X receptor homologue Ultraspiracle (USP) [21]. It can regulate the downstream primary genes (*E75*, *Br-C*, *E74* and *E93*) [22] and secondary response genes (*HR3*, *HR4*, *HR*38 and *E78*), and then regulate the expressions of terminal genes through *FTZ*-f1 gene [23]. In *Drosophila*, the transcript levels of *Phm* and *DIB* dropped significantly with the loss of *FTZ*-f1 function in PG cells [24]. In *Daphnia magna*, the *Neverland*, *CYP314A1* and *CYP307A1* genes had been identified, and their functions had been analyzed [25–27]. However, the gene expression and functional analysis of *CYP302A1*, *CYP306A1* and *CYP315A1* in cladocera (including *Daphnia*) have not been explored.

As one of the key genes in insect ecdysone synthesis, *CYP302A1* that can catalyze the carbon-22 hydroxylase is a member of the mitochondrial cytochrome P450 family [28–31]. Chavez et al. (2000) found that inactive ecdysone and 20E had lower titers in the *CYP302A1* (*DIB*) mutant embryos of *Drosophila*, and two 20E-inducible genes (*IMP-E1* and *L1*) failed to express in some tissues, resulting in anaphase abnormality in morphology [13]. After RNAi in *Sogatella furcifera* and *Laodelphax striatellus*, the expression levels of both *CYP302A1* gene and ecdysone receptor (*EcR*) gene decreased significantly and the development and death time of nymphs delayed [31]. In spatio-temporal expression profiling of *Bombyx mori*, *CYP302A1* gene showed a higher expression in the ovary, testis and head of the larvae [32]. A few investigations have reported on the genes related to the ecdysone synthesis pathway of Cladocera [25–27, 33], but the molecular mechanisms of ecdysone synthesis pathway and ecdysone signal transduction pathway need still to be further revealed.

The study on molecular biology of *Daphnia* species has become a hot spot, with the successive reports on the genome of *Daphnia pulex* and *D. magna* [34, 35]. In this study, based on the transcriptome, real-time PCR and RNAi technologies, the *CYP302A1* gene cloning, and the changes of downstream response gene expressions and individual phenotypic characteristics after knock-down of the gene were analyzed in *D. sinensis*. Meanwhile, the function of the *CYP302A1* gene was discussed. Moreover, the expression sites of the *CYP302A1* gene in *D. sinensis* was also detected by whole mount in situ hybridization technique. Our results will help to clarify the ecdysone synthesis pathway of *Daphnia* species, and provide a reference for the future study of ecdysis-related signaling pathways.

## Results

### Sequence and phylogenetic analysis of *CYP302A1* gene

**Fig. 1:**
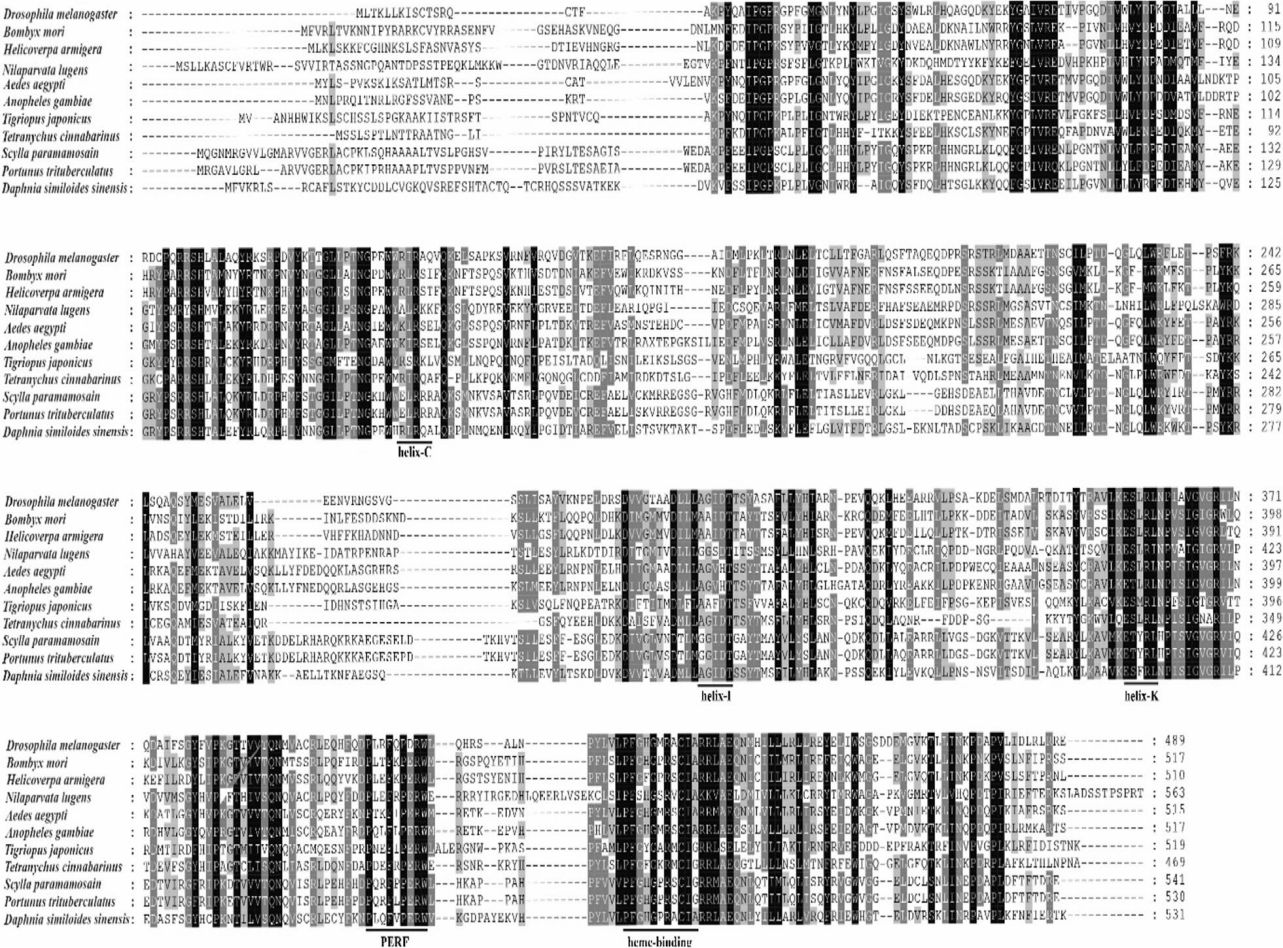
Homology comparison of amino acid sequences of the *CYP302A1* gene in *D. sinensis* with other arthropods. Note: Underlines are the conserved domain of helix-C, helix-I, helix-K, PERF and heme binding

**Fig. 2:**
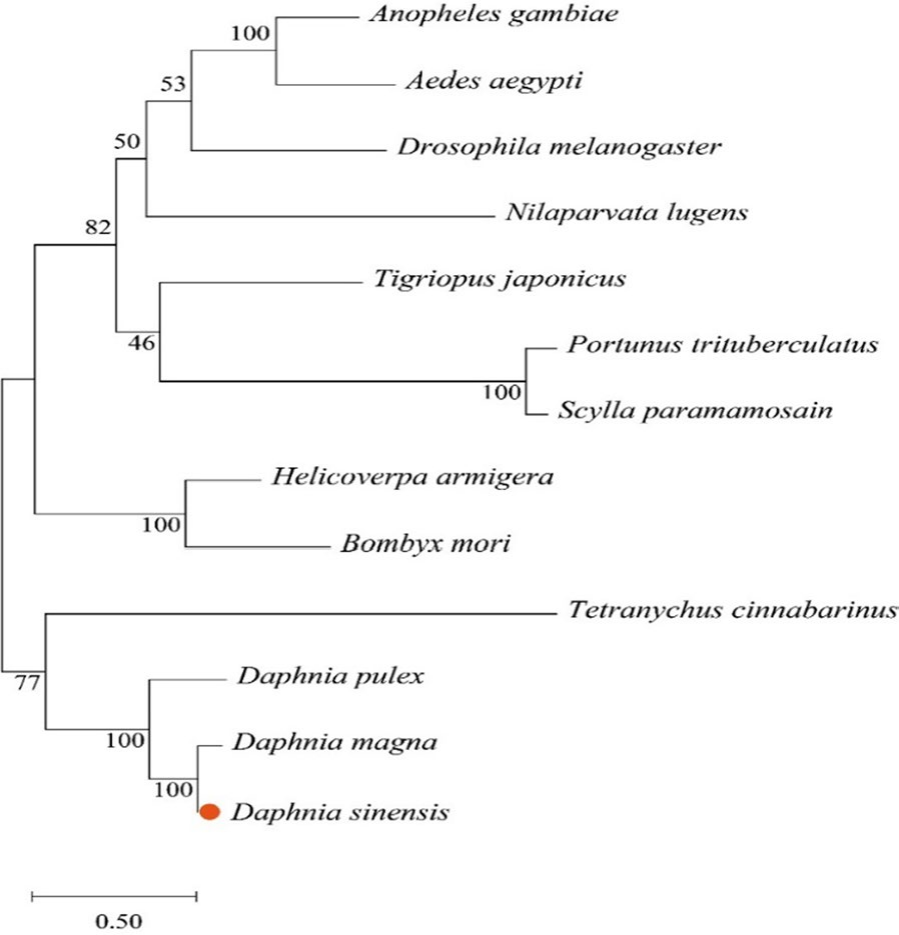
Phylogenetic trees of the *CYP302A1* gene in *D. sinensis*

The full-length of the *CYP302A1* gene in *D. sinensis* is 5926 bp with the open reading frame (ORF) of 1596 bp, which encodes 531 amino acids. The molecular formula of its protein is C_2743_H_4344_N_750_O_778_S_17_, with a molecular weight of 60.82 kDa and an isoelectric point of 9.29. Moreover, there is no signal peptide sequence and transmembrane domain in the *CYP302A1* gene. Compared with other arthropods, the *CYP302A1* gene of *D. sinensis* had the highest homology with *Tigriopus japonicus* (45.99%). In the amino acid sequence of the *CYP302A1* gene, there were five characteristic conserved domains (namely, helix-C, helix-K, helix-I, PERF and heme binding) (Fig. 1). The phylogenetic tree indicated that *D. sinensis* was the closely related to the two other *Daphnia* species *D. magna* and *D. pulex*, followed by *Tetranychus cinnabarinus* (Fig. 2).

### Induced expression of dsRNA

**Fig. 3:**
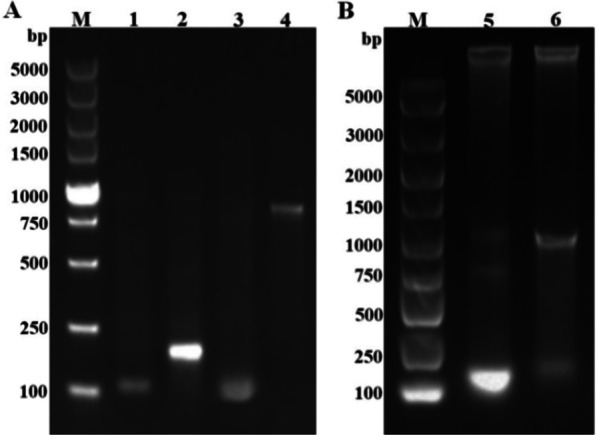
Induced expression of L4440-DIB and L4440-EGFP fragments. **A** Induced expression levels of HT115 strain carrying L4440-EGFP recombinant plasmid and L4440 vector plasmid; **B** induced expression level of HT115 strain carrying l4440-DIB recombinant plasmid; M: DNA molecular weight standard; 1: HT115 strain carrying L4440 vector plasmid was not induced; 2: induced product of HT115 strain carrying L4440 vector plasmid; 3: HT115 strain carrying l4440-EGFP recombinant plasmid was not induced; 4: induced product of HT115 strain carrying l4440-EGFP recombinant plasmid; 5: HT115 strain carrying l4440-DIB recombinant plasmid was not induced; 6: Induced product of HT115 strain carrying l4440-DIB recombinant plasmid

Usually, the expression fragments of empty body, L4440-EGFP (Enhanced Green Fluorescent Protein) recombinant plasmid and L4440-DIB recombinant plasmid induced by IPTG are 163 bp, 913 bp and 983 bp, respectively. 1% Agarose gel electrophoresis showed that the L4440 vector plasmid and L4440-EGFP were about 150 bp and 900 bp in size, respectively (Fig. 3A), and the L4440-DIB recombinant plasmid was about 1000 bp (Fig. 3B). Therefore, these results were consistent with the expected lengths of the plasmids.

### mRNA expression of the *CYP302A1* gene after RNAi

**Fig. 4:**
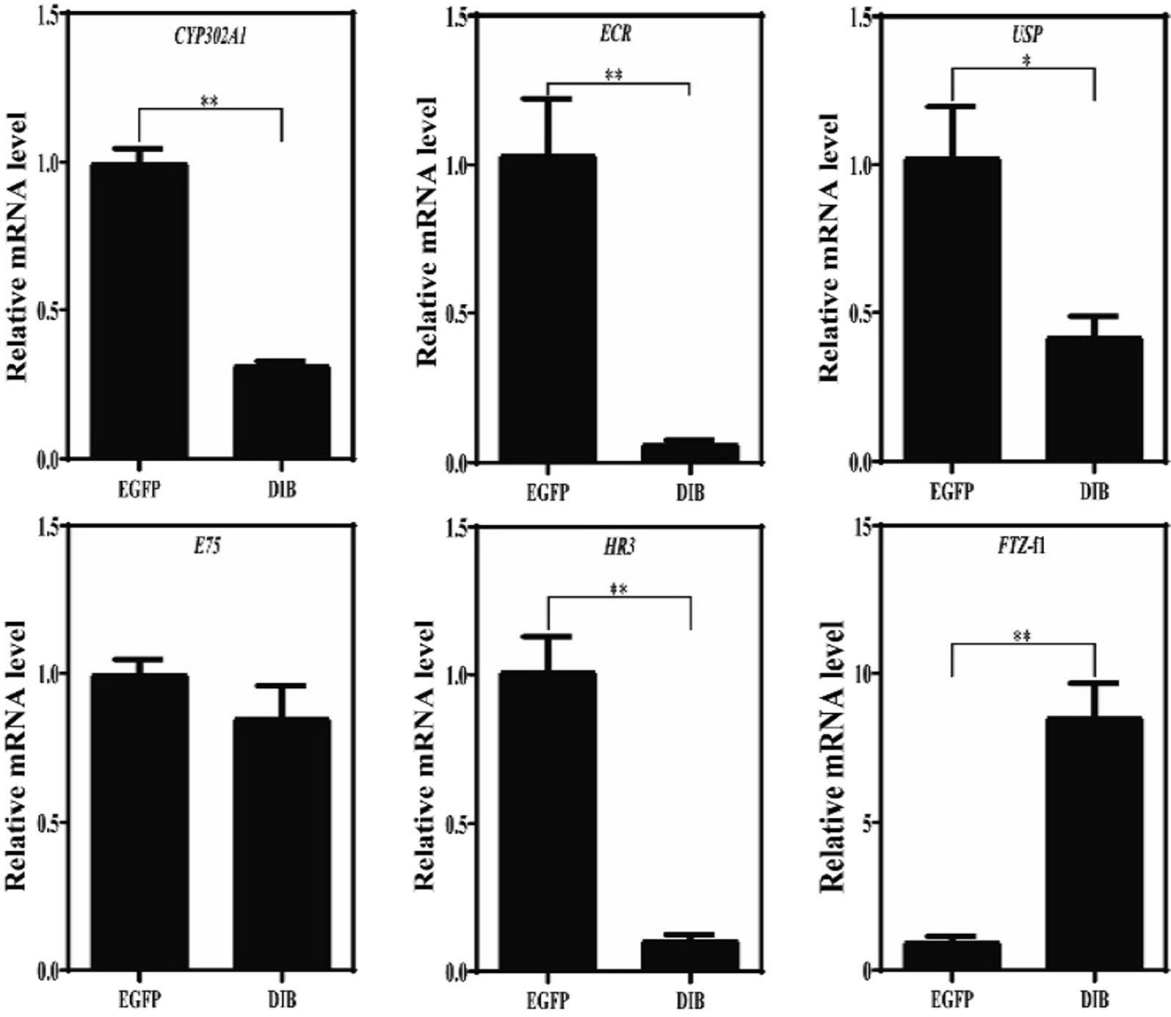
qPCR results of ecdysis gene *CYP302A1* and its downstream response gene in *D. sinensis* fed by 5% *E. coli* concentration. Note: E5-EGFP: 5% *E. coli* concentration containing L4440-EGFP; E5-DIB: 5% *E. coli* concentration containing L4440-DIB. *stands for *P* < 0.05; **stands for *P* < 0.01. EcR: ecdysone receptor; USP: Ultraspiracle; E75: ecdysone-induced protein 75, which is a nuclear hormone receptor; HR3: hormone receptor 3; FTZ-f1: Fushi-tarazu factor 1, which is an ecdysone-inducible transcription factor. All these genes respond to the regulation of 20-hydroxyecdysone

**Fig. 5:**
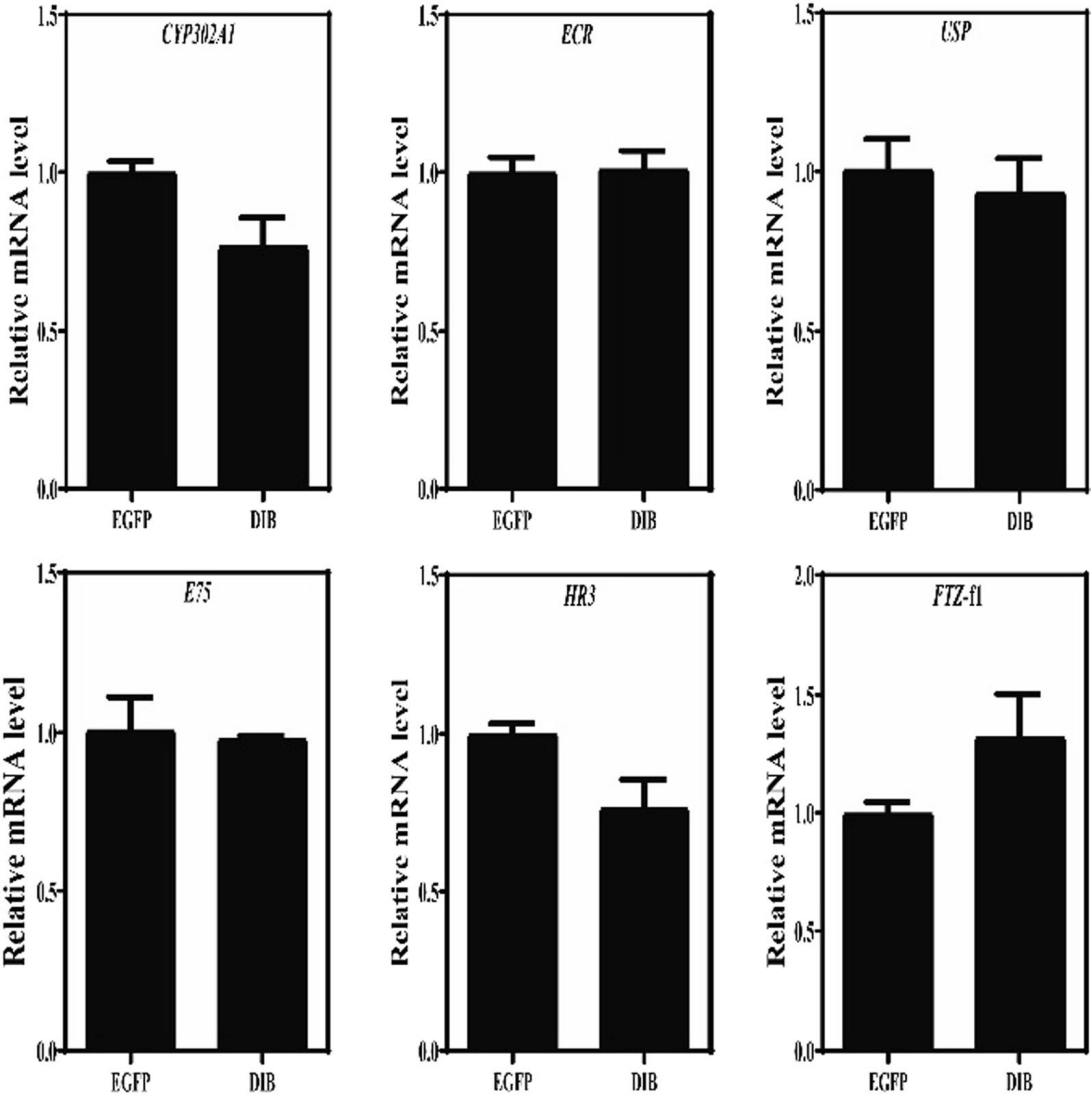
qPCR results of ecdysisgene*CYP302A1* and its downstream response gene in *D. sinensis* fed by 10% *E. coli* concentration. Note: E10-EGFP: 10% *E. coli* concentration containing L4440-EGFP; E10-DIB: 10% *E. coli* concentration containing L4440-DIB

Compared with the control treatment, the mRNA expression levels of the *CYP302A1*gene of *D. sinensis* in the 5% and 10% *E. coli* treatments decreased by 68.34% and 23.32%, respectively (Figs. 4, 5). Under the 5% *E. coli* concentration, the expression levels of *EcR*, *USP* and *HR3* genes in the downstream decreased significantly, whereas the expression levels of *FTZ*-f1 gene increased significantly (Fig. 4). Under the 10% *E. coli* concentration, there was no significant difference between E10-DIB treatment and E10-EGFP treatment. Moreover, the expression levels of *USP* and *HR3* genes in the downstream decreased whereas that of the *FTZ*-f1 gene increased, however, no significant differences were observed (Fig. 5). These results indicated that the dsRNA-DIB containing 5% *E. coli* concentration inhibited significantly the expression level of the *CYP302A1* gene in *D. sinensis* whereas the interference efficiency was low under higher *E. coli* concentration (10%).

### Phenotypic changes of *D. sinensis* after RNAi

**Fig. 6:**
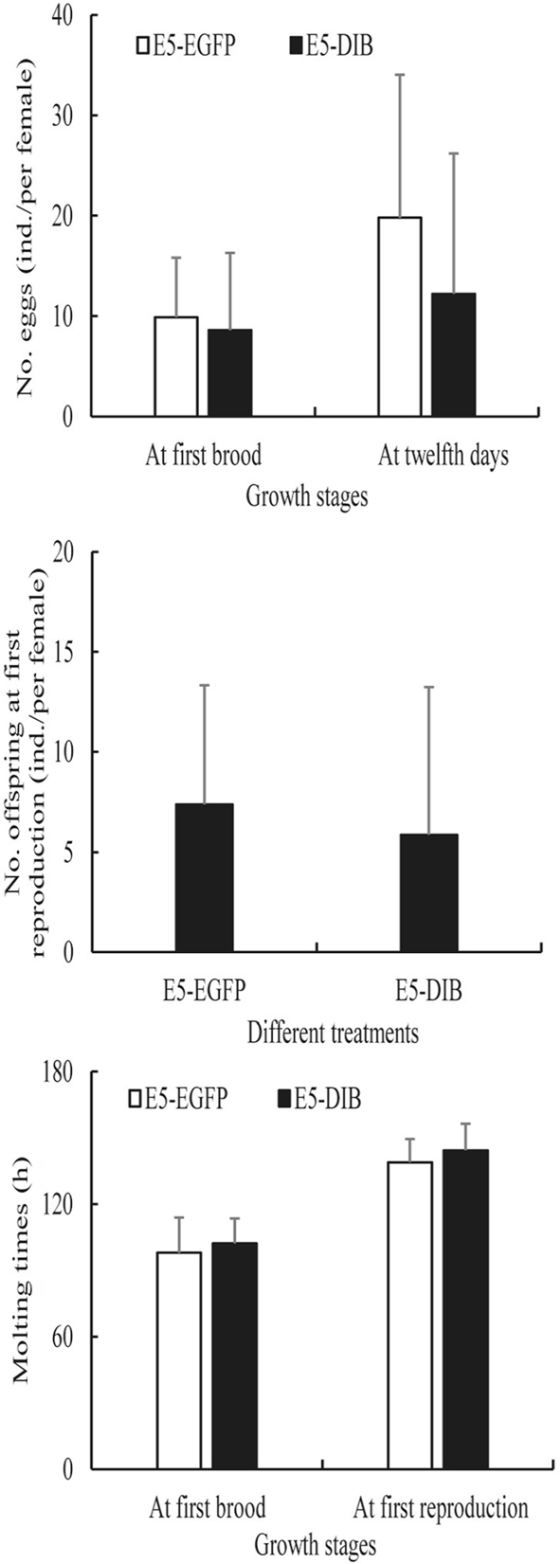
No. offspring at first reproduction, and no. eggs and molting time at two growth stages of *D. sinensis* after RNAi

Both no. eggs at first brood and no. offspring at first reproduction of *D. sinensis* in the E5-EGFP treatment were bigger than those in the E5-DIB treatment. However, no. eggs at first brood of *D. sinensis* at the 12th day of the experiment in the E5-EGFP treatment was significantly bigger than one in the E5-DIB treatment (Fig. 6). The molting time at first brood of *D. sinensis* in the E5-EGFP treatment were shorter than those in the E5-DIB treatment as well as the molting time at first reproduction (Fig. 6).

### Positioning analysis of the *CYP302A1* gene in *D. sinensis*

**Fig. 7:**
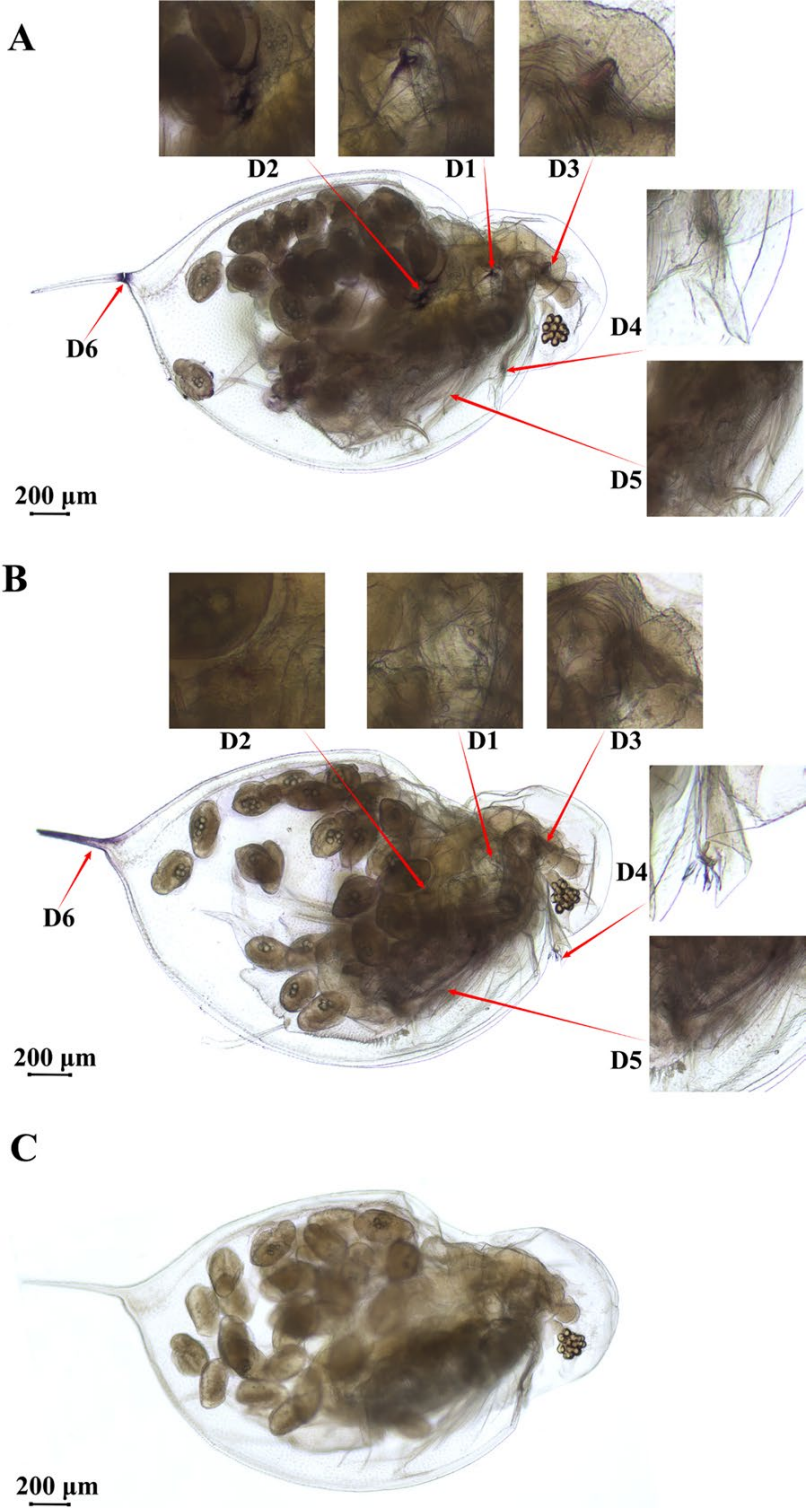
Expression sites of the *CYP302A1* gene in *D. sinensis*. Blue is the positive signal; **A** in situ hybridization map of *D. sinensis* without RNAi (antisense probe); **B** in situ hybridization map of *D. sinensis* after RNAi (antisense probe); **C** negative control (sense probe); D1: first antennal nerve; D2: ovary; D3: cecae; D4: olfactory hair; D5: thoracic limb; D6: tail spine

Whole mount in situ hybridization showed that the *CYP302A1* gene in *D. sinensis* had six expression sites, which respectively located in the first antennal ganglion, ovary, cecae, olfactory hair, thoracic limb and tail spine (Fig. 7A). After RNAi, the expression signal of the *CYP302A1* gene disappeared in the first antennal ganglion of *D. sinensis*, and the expression signal at the ovary was also greatly attenuated (Fig. 7B). Similarly, the expression sites of the *CYP302A1* gene in *D. sinensis* was not detected in the negative control experiment (Fig. 7C).

## Discussion

Ecdysone is synthesized under the catalyzation of a series of cytochrome P450 family coding enzymes in arthropods, which can regulate basic physiological processes such as molting and reproduction in arthropods [14, 29, 33]. The cytochrome P450 family is an ancient gene family that exists in almost all organisms [38, 39]. Although the amino acid sequences of the cytochrome P450 family member have high variability, a certain conservative domain are still found. In insects, conserved domains include helix-C, helix-I, helix-K, PERF and heme-binding [13]. In this study, the *CYP302A1* gene of *D. sinensis* contained also the above five conserved domains, indicating that *CYP302A1* gene belonged to the cytochrome P450 family.

In recent years, with the development of RNAi technology [40], RNAi has been widely used in the study of biological gene function [41, 42]. Using chitin synthase gene A (*SeCHSA*) as the target gene, the growth and development of *Spodoptera exigua* larvae fed by *E. coli* containing dsRNA of *SeCHSA* was disturbed, and then the mortality rates in the 5th instar larvae increased significantly [43]. After either feeding or injecting dsRNA of the sex-determining gene *Transformer-2* to *Zeugodacus scutellata*, the *Transformer-2* gene were all silenced, and increased significantly the number of male among their offspring [44]. Through RNAi to the appendage terminal *Distal-less* (*Dll*) gene of *D. magna*, it was found that the appendage terminal was deficient [45]. In this study, the expression levels of the *CYP302A1* gene decreased significantly (knock-down of 68.34%) in the 5% *E. coli* treatment, whereas it was only knock-down of 23.32% in the 10% *E. coli* treatment, indicating that the silencing effect at the lower *E. coli* concentration was better than at higher concentration. This phenomenon was also observed in other *Daphnia* species [46, 47]. Through the feeding experiment on different concentrations of *E. coli* expressing the phenoloxidase dsRNA, Schumpert et al. (2015) found that the overall % with clear carapace (20%) at the end of the experiment of *Daphnia melanica* under higher concentration was lower than one (60%) under lower concentration [46]. After the 14-days feeding of dsRNA-Dhb2, Eytcheson and LeBlanc (2018) found also that the mRNA levels of *Dhb2* of *D. magna* under higher *E. coli* concentration was significantly lower than one under lower concentration [47]. Therefore, it is likely that more bacteria consumption under higher *E. coli* concentration may have resulted in a decrease of dsRNA delivery and attenuation of siRNA suppression in *D. sinensis*. Moreover, the optimal concentration and time of silencing target genes can depend on different experimental animals or genes. After injecting dsRNAs of *CYP307A2* and *CYP314A1* genes, the development of the ovaries in female adults of *Agasicles hygrophila* delayed, and the egg production dropped significantly, and the expression level of vitellogenin gene (*Vg*) down-regulated significantly [48]. Similarly, when injected with dsRNA of the *CYP315A1* gene, the adult ovary in *Plutella xylostella* became smaller and mature eggs decreased, and the cumulative number of eggs also decreased significantly [49]. In this study, the expression levels of the ecdysone receptor *EcR* gene and the *USP* gene in *D. sinensis* belonging to the downstream response genes of ecdysone decreased significantly after RNAi. Moreover, the knock-down of the *CYP302A1* gene resulted in a significant decrease in the expression level of the downstream *HR3* gene, but no significant effect on *E75* gene was observed. During the experiment, the destruction of these downstream genes could result in some aborted eggs or dead embryos of *D. sinensis* in the incubation capsule. Hannas and Leblanc (2010) also found that the ecdysone could significantly affect the expression level of *HR3* gene but less effect on *E75* gene [50]. In this study, the knock-down of the *CYP302A1* gene increased significantly the expression level of the *FTZ*-f1 gene in *D. sinensis*. Usually, *FTZ-f1* is mainly responsible for regulating the expression of upstream *CYP302A1*, *CYP306A1* and *CYP315A1* genes in *Drosophila* ecdysone signal transduction [30]. Therefore, our results strongly supported that the *CYP302A1* gene is an ecdysone synthesis pathway gene in *D. sinensis*, affecting the molting and reproduction of *Daphnia*.

Rewitz et al. (2006) found that the ecdysone synthesis pathway gene *CYP302A1* of the tobacco hawkmoth was mainly expressed in the prethymocytes during the larval stage whereas it was detected in the fat body, midgut, ganglia, malpighian tubules and epidermis in animals after the fifth ecdysis [51]. In this study, the *CYP302A1* gene in *D. sinensis* had mainly six expression sites, which located in the first antennal ganglion, ovary, cecae, olfactory hair, thoracic limb and tail spine. However, only expression signal of the *CYP302A1* gene in the first antennal ganglion of *D. sinensis* disappeared after RNAi. Moreover, the expression signals in the ovary weakened greatly. Usually, the first antennae are the sites of signalling for the hormonal induction of reproduction of cladocera. Therefore, the *CYP302A1* gene can be involved in the reproductive transformation of *D. sinensis*. It was also consistent with the aborted eggs or dead embryos in the incubation capsule of *D. sinensis* under the 5% *E. coli* concentration. Sumiya et al. (2014, 2016) found that both *Neverland1* and *CYP314A1* are involved in the synthesis of ecdysone in *D. magna*, and intestinal epithelial cells were responsible for this synthesis [25, 26]. Usually, the cecum is located in the left and right sides of the front end of the midgut in cladocera, with a pair of ear-like appendages. In this study, the expression site of the *CYP302A1* gene at the cecae of *D. sinensis* is consistent with other studies [25, 26]. It was likely that the cecum in *D. sinensis* was an important synthesis and secretion site of ecdysone, and some sites of the thoracic limb and tail spine began to express the *CYP302A1* gene during the ecdysis. In conclusion, the *CYP302A1* gene in *D. sinensis* was a gene related to synthesis of the ecdysone, which would play an important role in the molting and reproduction of cladoceran.

## Conclusions

Molting is an important physiological process in the life history of cladocera, which is mainly regulated by juvenile hormone and ecdysone. *CYP302A1* is the key enzyme which plays a critical role in the synthesis of ecdysone of insects, but it has not been identified in cladocera. In this study, the *CYP302A1* gene was also found in *D. sinensis*. The amino acid sequence analysis revealed that the *CYP302A1* gene of *D. sinensis* had five characteristic conserved regions of cytochrome P450 family, namely, helix-C, helix-K, helix-I, PERF and heme-binding. In dsRNA mediated experiment, the expression level of the *CYP302A1* gene decreased significantly in the 5% *E. coli* treatment. Meanwhile, the expression levels of *EcR*, *USP* and *HR3* genes in the downstream decreased also significantly whereas that of *FTZ-f1* gene increased significantly. Moreover, the development of embryos in the incubation capsule of *D. sinensis* appeared abnormal or disintegrated. The whole-mount in situ hybridization indicated that the *CYP302A1* gene of *D. sinensis* had six expression sites (namely the first antennal ganglion, ovary, cecae, olfactory hair, thoracic limb and tail spine) before RNAi. However, the expression signal of the *CYP302A1* gene of *D. sinensis* disappeared in the first antennal ganglion and obviously attenuated in the ovary after RNAi. Our results suggested that the *CYP302A1* gene could play an important role in the ecdysone synthesis pathway of *D. sinensis*.

## Materials and methods

### *D. sinensis* culture

*D. sinensis* were obtained from the hatching of resting eggs in the sediments of Lake Chaohu, China. The individual was monoclonally cultured in an intelligent light incubator at 25 °C, with a 12 h:12 h light/dark cycle. The culture medium was changed every day, and *D. sinensis* were fed with 2 × 10^5^ cells/mL of *Tetradesmus obliquus*. The culture medium was filtered and aerated tap water over 48 h.

### RNA extraction and first-strand cDNA synthesis

50 female adults of *D. sinensis* were collected and stored in 100 μL RNAlater (Biosharp, Hefei, China) in 1.7 mL tubes, and total RNA were extracted by the MiniBEST universal RNA kit (TaKaRa, Dalian, China). The quality and purity of RNA was measured using a NanoDrop spectrophotometer (MD2000D, Biofuture, England) and Agarose electrophoresis. The first-strand cDNA was synthesized using the PrimeScript™RT kit (TaKaRa) according to the manufacturer’s instructions, and then stored at − 80 °C.

### Sequence and phylogenetic analysis of *CYP302A1* gene

The full-length *CYP302A1* gene was obtained by sequencing, splicing and functional annotation of the *D. sinensis* transcriptome in our previous investigations [52]. The open reading frame of the nucleotide sequence of the *CYP302A1* gene was analyzed using the online prediction tool ORF finder (https://www.ncbi.nlm.nih.gov/orffinder), and the amino acid sequence was obtained. The amino acid deduction analysis and alignment of the *CYP302A1* gene were performed by DNAMAN software, and the phylogenetic tree was constructed by MAGA 11.0 software. Isoelectric point analysis were executed using ExPASy ProtParam (https://web.expasy.org/protparam). Signal peptide and transmembrane region in protein were respectively predicted using Signal 4.1 Server (http://www.cbs.dtu.dk/services/SignalP-4.1) and TMHMM (http://www.cbs.Dtu.dk/services/TMHMM).

### Induced expression of dsRNA

The primers were designed according to the transcriptome data and *EGFP* (Enhanced Green Fluorescent Protein) plasmid sequence (Table 1). *EGFP* was used as a negative control [46, 53]. The PCR program was as follows: 95 °C for 3 min, 95 °C for 15 s, 55–60 °C for 15 s, and 72 °C for 40 s, followed by 35 cycles, and 72 °C for 5 min. PCR products were detected by a 1% agarose gel electrophoresis. PCR products of *DIB/EGFP* were subcloned into the pEASY-Blunt3 cloning vector (TransGen, Beijing, China) and sequenced (General Biol, Nanjing, China). After sequencing, the expression vectors L4440 and pEASY-Blunt3-DIB/EGFP plasmid were digested using restriction enzymes *BamH* I and *Xho* I (TaKaRa), and then ligated. The L4440 vector contains two T7 promoters which can be induced by isopropyl β-d-1-thiogalactopyranoside (IPTG) to produce dsRNA of the sequence ligated between these promoters. The L4440 constructs were transformed into *E. coli* DH5α cells (Sangon Biotech, Shanghai, China), and the vector was confirmed by sequencing (General Biol). After sequencing, the L4440-DIB plasmid was transformed into *E. coli* HT115 cells (a strain deficient in RNase III and an efficient production for dsRNAs). The transformed cells were cultured overnight in LB medium containing ampicillin (100 μg/mL, Sangon Biotech) and tetracycline (12.5 μg/mL, Sangon Biotech) for the *CYP302A1* RNAi experiments. Isopropyl IPTG (1.0 mM, Sangon Biotech) was added to induce the T7 RNA polymerase and subsequent production of dsRNA of the target sequence. The expression of dsRNA was detected by 1% agarose gel electrophoresis. The primers used in the experiments were listed in Table 1.

**Table 1:** Names and sequences of primers used in the experiment

Primer name	Forward (5′–3′)	Reverse (5′–3′)
*DIB*	CGCGGATCCGAAGCGACTAATGCAATCGC	CCGCTCGAGTTCGGGACCGTTTGTTGGA
*EGFP*	CGCGGATCCATGGTGAGCAAGGGCGAGG	CCGCTCGAGTTACTTGTACAGCTCGTCCATGCCG
*qDIB*	ATACTTCGGACGGATAATG	CAACGCAATACTCTCAATG
*qEGFP*	CGCACCATCTTCTTCAAG	GTGGCTGTTGTAGTTGTAC
*EcR*	GAGGCGCTGCAGGCTTAC	GAGTTTGGCAAACTCCGTCATC
*USP*	GTTGGAGTCAAGGATGGTATCGT	AGCCGAGTTCCGGTGGAT
*E75*	TCCGGAGAAGTATTCAACAAAAGA	TGCGAAGAATGGAGCACTGT
*HR3*	AGTCATCACCTGCGAGGGC	GAACTTTGCGACCGCCG
*FTZ-*f1	ATCGTGCAAGGGATTCTTCA	ATCAGCGACGCAAGAATAGG
*GAPDH*	TCGTCTCCAATGCTTCTT	CGGTCCATCAACAGTCTT

*DIB* and *EGFP* are interference primer, with length of the amplicons is respectively 820 bp and 750 bp. The other primers are qPCR primer, and the underlines of forward and reverse primer are the restriction endonuclease sequence

### RNAi feeding protocol

Four food treatments were selected for the experiments, namely, E5-EGFP: 5% dsRNA-EGFP + 95% *T. obliquus*; E5-DIB: 5% dsRNA-DIB + 95% *T. obliquus*; E10-EGFP: 10% dsRNA-EGFP + 90% *T. obliquus*; E10-DIB: 10% dsRNA-DIB + 90% *T. obliquus*. There were three replicates at each food treatment. Total food biomass was 20 mg/L wet weight. 15 animals (birth time < 12 h) in each replicate were employed as the mother. During the experiment, the culture medium at each food treatment was refreshed every day, and all newborns produced by the mother were immediately removed. All mothers in each replicate were collected at the twelfth day after feeding, placed in a 1.7 mL tube containing 100 μL RNAlater and stored in a refrigerator at 4℃ for 12 h, and then transferred to an ultra-low temperature refrigerator at − 80℃. After RNAi, the expression levels of related genes were determined by qPCR, and the relative expression levels of target genes was calculated by 2^−△△^Ct. The life history parameters of the four growth stages (at birth, at first brood, at first reproduction and at the twelfth days) were observed and recorded during the experiment.

### Whole mount in situ hybridization

In order to prepare probes for in situ hybridization according to the ORF of the *CYP302A1* gene, the sequences of specific primers were designed as follows: ISH-DIB-Forward: CGCGGATCCGAGCTTTATACTGTATCATCTTGCC, ISH-DIB-Reverse: CCGCTCGAGGACTCTTTTACTGCAGCCTTTAGAT, which the length of the amplicon is 150 bp. Target fragment was synthesized according to the primer sequence. After sequencing, the positive clone bacteria were amplified and cultured, and then the Blunt3-ISH-DIB vector plasmid was extracted. The concentration and purity of the plasmid were determined by a NanoDrop spectrophotometer (MD2000D, Biofuture). The linearized plasmid was obtained through restricted digestion of *BamH* I or *Xho* I, and the digested DNA fragments were purified and used as templates for sense and antisense probes, respectively. RNA probes were synthesized through DIG RNA Labeling Kit (SP6/SP7) (Roche, Mannheim, Germany), and then digested the probe cDNA template using DNase (RNase-free). Probe synthesis system (20 μL): linear plasmid ≤ 1 μg, 10 × NTP labeling mixture 2 μL, 10 × transcription buffer 1 μL, RNase Inhebitor 1 μL, RNA Polymerase SP6/T7 2 μL, and then added to 20 μL with RNase-free H_2_O. In addition, a 1/9 volume of 5 M LiCl and 2 volumes of absolute ethanol were added, and were incubated overnight at − 20 °C. RNA pellets was washed twice with 75% ethanol, and then dried to remove residual ethanol. Finally, RNA pellets was re-suspended in 30 μL diethylpyrocarbonate water, which 1 μL RNA inhibitor (20 U) were added. Aliquots of RNA solutions (1 µL) were added and electrophoresed, and the concentrations were measured. Remaining RNA probes were stored at − 20 °C.

According to the RNAi feeding protocol, 50 female adults of *D. sinensis* were collected. All samples were fixed in 4% paraformaldehyde (PFA) overnight, and then were replaced by anhydrous methanol and remained at − 20 °C. Whole-mount in situ hybridization was carried out according to previously published methods [36, 37] with some modifications. The specimens stored at − 20 °C were rehydrated gradually with methanol-PBST and digested with proteinase K (10 µg/mL, Solarbio, Beijing, China). The individuals were digested at 37 °C for 12 min. After pre-hybridization at 68 °C for 2.5 h, 100 µL RNA probe which was diluted 1:100 was added and incubated at 70 °C overnight. The specimens were blocked for approximately 2 h at room temperature with slow shaking in MAB block solution, and then added anti-DIG antibody (diluted 1:5000, Roche) and incubated at 4 °C for 13 h. Finally, antibody solution was discarded and the specimens were washed in MABT. At room temperature, the NBT liquid dye (Roche) was used to shade the color for 15 min − 2h, and then the individuals were fixed in 4% PFA for 20 min. Hybridization was observed with a fluorescence microscope (Olympus, CX21).

## Data Availability

All data generated or analyzed during this study included in the published article and its additional files.
